# The impact of a brief web-based intervention on improving awareness of the need to reduce alcohol consumption during pregnancy among high-risk female college student drinkers: a quasi-experimental study

**DOI:** 10.4069/whn.2025.11.19

**Published:** 2025-12-31

**Authors:** Hae Won Kim, Saem Yi Kang

**Affiliations:** 1 Research Institute of Nursing Science, Center for Human-Caring Nurse Leaders for the Future by Brain Korea 21 (BK 21) Four Project, Seoul, Korea; 2 College of Nursing, Seoul National University, Seoul, Korea; 3 College of Nursing, Gachon University, Inchon, Korea

**Keywords:** Alcohol drinking, Health behavior, Internet-based intervention, Preconception care

## Abstract

**Purpose:**

This study aimed to assess the impact of a brief web-based intervention for female college students with high-risk drinking on current drinking habits, as well as future intention and awareness regarding abstaining from alcohol during pregnancy.

**Methods:**

This quasi-experimental study recruited female college students with high-risk drinking and assigned them to either an intervention group (n=23) or a control group (n=23) based on availability and preference. The intervention group received a brief web-based session (30 minutes) focused on alcohol use prevention for future pregnancies and monitored their daily drinking using a mobile app for 1 month. Outcome measures included the Alcohol Use Disorders Identification Test, variables derived from the Theory of Planned Behavior (attitude, subjective norms, and intention) related to pregnancy, drinking refusal self-efficacy, and alcohol outcome expectancies. Data were analyzed using repeated-measures analysis of variance (ANOVA).

**Results:**

There were significant changes in the mean differences from baseline scores between the groups with respect to positive attitudes toward drinking during pregnancy (t=−2.59, *p*=.013) and intentions to abstain from drinking during pregnancy (t=2.35, *p*=.005). Repeated-measures ANOVA demonstrated a significant interaction between group and time for both attitude (F=6.69, *p*=.013) and intention (F=8.58, *p*=.005). In addition, a significant improvement in drinking refusal self-efficacy was observed (t=2.49, *p*=.016).

**Conclusion:**

The brief web-based intervention significantly improved attitudes toward drinking during pregnancy, intentions to abstain from drinking during pregnancy, and drinking refusal self-efficacy, highlighting both the effectiveness and importance of this intervention.

## Introduction

National data indicate that women in their 20s and 30s have the highest prevalence of high-risk drinking, representing a substantial public health concern among reproductive-aged women [1]. A previous study examining alcohol consumption among South Korean college students found that 72.9% of female students reported monthly drinking, exceeding the 64.1% observed among women aged 19 to 29 years. In addition, high-risk drinking was reported in 17.2% of female college students, nearly double the corresponding rate of 9.6% among women aged 19 to 29 years [2].

It is widely acknowledged that alcohol consumption can result in adverse fetal developmental outcomes [3]. Even prior to pregnancy, alcohol use among women of reproductive age significantly increases the likelihood of infertility treatment and negatively affects fertility [4,5]. A recent mouse model study further demonstrated that prepregnancy alcohol consumption can induce maternal metabolic dysfunction and increase the risk of fetal developmental abnormalities, birth defects, and macrosomia [6].

Despite these well-documented risks, many female college students demonstrate limited awareness of the dangers associated with alcohol use before conception or during early pregnancy, including fetal alcohol spectrum disorders (FASD) [7,8]. This persistent gap in awareness highlights the urgent need for early, targeted interventions for women of reproductive age.

Women of reproductive age who engage in high-risk drinking should consider reducing alcohol consumption in preparation for future pregnancies. Previous research has demonstrated a significant association between prepregnancy alcohol use, high-risk drinking patterns, and alcohol consumption during pregnancy [9,10]. Moreover, a woman’s current alcohol consumption pattern has been identified as a predictor of her intention to drink alcohol during a future pregnancy [11].

A previous study found that female college students scoring 8 or higher on the Alcohol Use Disorders Identification Test (AUDIT), indicative of high-risk drinking, were less likely to intend to abstain from drinking during pregnancy than peers who did not engage in problem drinking [12]. Accordingly, the present study emphasizes the importance of early intervention to prevent alcohol use during pregnancy and to encourage reductions in alcohol consumption among women exhibiting high-risk drinking behaviors.

The theory of planned behavior (TPB) [13] provides a robust theoretical framework for understanding alcohol-related behaviors [14]. Interventions grounded in TPB have been shown to reduce overall alcohol consumption and binge drinking among newly enrolled university students [15]. Consistent with established guidance for developing TPB-based interventions [16], we examined awareness related to alcohol consumption during pregnancy among female college students using TPB constructs. Attitudes, subjective norms, and drinking frequency have been shown to influence intentions to abstain from alcohol during pregnancy [12].

To enhance the explanatory power of TPB, additional determinants influencing intention or behavior may be incorporated beyond the core constructs [17]. Self-efficacy, defined as an individual’s belief in their ability to perform a specific behavior [18], is conceptually related to, but distinct from, perceived behavioral control (PBC), and has demonstrated a stronger association with intention than PBC [14,19]. Drinking refusal self-efficacy reflects perceived ability to decline alcohol in specific situations and has been associated with lower frequency and quantity of alcohol consumption [20]. Furthermore, drinking refusal self-efficacy mediates the relationship between alcohol outcome expectancies and alcohol consumption [21]. Both drinking refusal self-efficacy and alcohol outcome expectancies significantly explain drinking behaviors among college students [22], and prior interventions have demonstrated improvements in drinking refusal self-efficacy [23]. Within the TPB framework, drinking refusal self-efficacy may be conceptualized as a functional extension or operationalization of PBC in the context of alcohol use. Enhancing self-efficacy thus represents a key mechanism through which interventions may influence intention and subsequent drinking behavior. Accordingly, the intervention effect in this study was examined across multiple factors, including attitudes, subjective norms, intention (TPB components), alcohol outcome expectancies, drinking refusal self-efficacy, and AUDIT scores representing current alcohol consumption.

Therefore, this study aimed to examine the impact of a brief web-based intervention on both current drinking behaviors and future intentions to abstain from alcohol during pregnancy.

The research hypotheses were as follows:

Hypothesis 1. Participants in the intervention group will show a greater decrease in AUDIT scores than those in the control group.

Hypothesis 2. The intervention group will exhibit more negative attitudes toward alcohol consumption during pregnancy than the control group.

Hypothesis 3. The intervention group will report more negative subjective norms related to alcohol use during pregnancy than the control group.

Hypothesis 4. The intervention group will demonstrate a stronger intention to abstain from alcohol during pregnancy than the control group.

Hypothesis 5. The intervention group will report lower positive alcohol outcome expectancies than the control group.

Hypothesis 6. The intervention group will report higher drinking refusal self-efficacy than the control group.

## Methods

**Ethics statement:** This study was approved by the Institutional Review Board of Seoul National University (No. 2008/003-019). Written informed consent was obtained from all participants prior to data collection. Participants received an electronic copy of the consent form in PDF format, signed it, and returned the signed document to the researcher before completing the survey and participating in the intervention.

### Study design and settings

**Figure 1.**
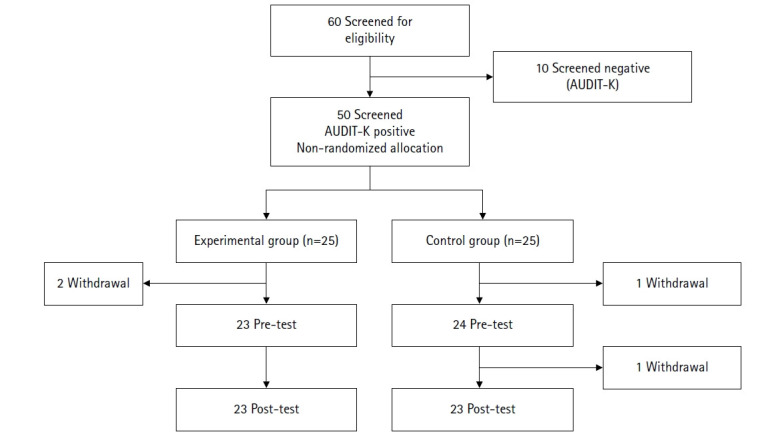
Study flow diagram following the TREND statement. AUDIT-K, Korean version of the Alcohol Use Disorders Identification Test.

A quasi-experimental pretest–posttest design was employed to assess the impact of a web-based program designed to increase intention to abstain from drinking during pregnancy among female college students engaged in high-risk drinking. This study adhered to the TREND (Transparent Reporting of Evaluations with Nonrandomized Designs) guidelines for reporting nonrandomized evaluations [24]. The research was conducted at Seoul National University located in Seoul, Korea. A flowchart illustrating the research design is presented in Fig. 1. Participants were assigned to one of two groups based on their availability and preference: the intervention group, which received a single educational intervention and self-monitored drinking behavior using a drinking record application for 1 month.

### Participants

From April to June 2021, female college students were recruited using convenience sampling through a campus-wide online platform that posted recruitment information and required college student authentication for access. Students identified as high-risk drinkers, defined as having an AUDIT score of ≥8, were eligible for participation. Exclusion criteria included prior participation in any educational program aimed at preventing alcohol consumption before or during pregnancy.

### Sample size estimation

The required sample size was calculated using G*Power version 3.1 (Heinrich Heine University Düsseldorf, Düsseldorf, Germany) for repeated-measures analysis of variance (ANOVA) examining a within–between interaction with two groups and two measurement points, consistent with the study’s objective of assessing between-group differences in changes over time in intention to reduce alcohol consumption during pregnancy. Based on previous web-based intervention studies among university students reporting small-to-moderate effects on intention [15], an effect size of f=0.27 was assumed for the group×time interaction. The significance level (α) was set at .05, and statistical power (1–β) was set at .95. This calculation indicated that a minimum total sample size of 48 participants (24 per group) was required. In the present study, 46 participants (23 per group) completed both the baseline and 4-week follow-up assessments and were included in the final analysis.

### Procedures and ethical considerations

For individuals who were screened and expressed interest in participation, the researcher explained the purpose of the study and principles of privacy and confidentiality related to voluntary participation via text message prior to enrollment. Participants were assigned to groups based on their willingness to participate, and baseline characteristics were compared to enhance group equivalence. For the intervention group, the researcher delivered the web-based intervention using the Zoom video conferencing system (Zoom Video Communications, Inc., San Jose, CA, USA) and informed participants that sessions were not recorded. Automatic recording and storage functions within Zoom were disabled. Participants were required to provide signed electronic informed consent in PDF format, and access to the web-based sessions was restricted to individuals who received encrypted access links. The control group did not receive any intervention, whereas the intervention group participated in a brief 30-minute web-based session. Data collection was conducted at baseline and again 4 weeks after the intervention. All data were collected anonymously and stored in a secure, password-protected database accessible only to the research team.

### Study intervention

#### The web-based brief intervention

We developed a single 30-minute web-based brief intervention session that integrated the theoretical framework of the TPB with practical strategies derived from motivational interviewing. This approach was chosen based on review studies demonstrating that brief, single-session alcohol interventions using motivational interviewing techniques are effective in significantly reducing alcohol use among heavy-drinking college students [25]. The content of the intervention was validated by four experts specializing in nursing and women’s health nursing. The intervention consisted of four components, each mapped to relevant TPB-related constructs: (a) assessing personal drinking habits, serving as a reflective activity to enhance self-awareness and initiate intention formation; (b) understanding the negative outcomes of alcohol use during pregnancy, aimed at influencing attitudes through evidence-based risks and health impacts; (c) recognizing the negative consequences of alcohol consumption for women of reproductive age, addressing both attitudes and subjective norms; and (d) setting drinking goals, designed to strengthen behavioral intention and indirectly support drinking refusal self-efficacy by promoting realistic planning and goal setting.

These four components were delivered through a real-time online lecture lasting 30 minutes using the Zoom platform. Participants were encouraged to develop plans to reduce alcohol consumption and to form an intention to abstain from drinking during pregnancy.

After the intervention, participants were instructed to monitor their daily alcohol consumption using a commercially available Korean smartphone drinking diary application (Sulchedule, CodersHigh Co., Ltd., Seoul, Korea) for 1 month. This application, available free of charge on both Android and iOS platforms, enabled users to record the type and quantity of alcohol consumed each day. Throughout the 1-month follow-up period, the researcher maintained contact with participants at 2-week intervals via phone calls or text messages to monitor changes in drinking behavior and to identify any challenges encountered in modifying alcohol consumption habits. In addition, participants were asked to submit screenshots from the application to the researcher as verification of daily alcohol tracking.

### Measurements

#### Problematic alcohol use (AUDIT)

Problematic alcohol use was assessed using the Korean version of the AUDIT [26], a screening instrument originally developed by the World Health Organization for the early identification of hazardous and harmful drinking [27]. The AUDIT consists of 10 items, each scored on a scale from 0 to 4, yielding a total possible score of 40. According to World Health Organization guidelines, a score of 7 or lower categorizes an individual as a non-problem drinker, whereas a score of 8 or higher indicates problematic drinking. The Korean version of the AUDIT has demonstrated good internal consistency, with Cronbach’s α values of .92 in validation research [26] and .85 in a previous survey study of female college students in Korea [12].

#### Attitudes, subjective norms, and intentions regarding alcohol consumption during pregnancy

Attitudes, subjective norms, and intentions regarding alcohol consumption during pregnancy were measured using TPB–based items adapted from previous studies [28], with permission obtained from the original author. Prior research applying TPB to drinking behavior has shown that PBC does not adequately predict drinking behavior [16,29]. In addition, a meta-analysis reported a small, negative, and non-significant association between PBC and drinking behavior [14]. Accordingly, this study employed only TPB components other than PBC. The instrument comprised (a) four attitude items, (b) three subjective norm items, and (c) three intention items, with each item scored on a scale of up to 4 points. Higher attitude scores (possible range, 4–20) reflect more positive or permissive attitudes toward drinking during pregnancy. Higher subjective norm scores (possible range, 3–15) indicate greater perceived social pressure to abstain from drinking during pregnancy. Higher intention scores (possible range, 3–15) represent stronger intentions to avoid alcohol in future pregnancies. The instrument demonstrated good reliability, with a Cronbach’s α of .86 [12].

#### Alcohol outcome expectancies

Alcohol outcome expectancies related to drinking were assessed using the positive expectancy subscale developed by Leigh and Stacy [30], which measures anticipated positive outcomes of alcohol consumption and has been translated into Korean [31]. This instrument consists of 19 items rated on a 5-point Likert scale, resulting in a total score range of 19–95. Higher scores indicate stronger expectancies of positive outcomes associated with alcohol use. In previous research, this scale demonstrated good internal consistency, with a Cronbach’s α of .86 [31].

#### Drinking refusal self-efficacy

Drinking refusal self-efficacy was measured using the instrument developed by Aas et al. [32] and translated into Korean [33]. This scale consists of seven items, each scored from 1 to 5, with higher total scores (possible range, 7–35) indicating greater self-efficacy in refusing alcohol. The Cronbach’s α for this instrument was .93 in previous research [33].

#### Sociodemographic characteristics

Sociodemographic characteristics were collected from participants and included age, year in college, academic major, religion, monthly alcohol expenditure, age at drinking onset, and smoking status. These data were collected only once, at baseline.

### Data analysis

Data were analyzed using SPSS/WIN Statistics version 22.0 (IBM Corp., Armonk, NY, USA). General characteristics and primary study variables were examined using frequencies, percentages, means, and standard deviations. Group differences in baseline characteristics and outcome variables were assessed using chi-square tests and independent t-tests to confirm homogeneity between the intervention and control groups. The effectiveness of the intervention was evaluated using repeated-measures ANOVA to examine the main effects of time and group, as well as time×group interaction effects. Statistical significance was set at *p*<0.05 for two-sided tests.

## Results

### Baseline general characteristics and outcome variables

Participants had a mean age of 21.0±2.0 years. Approximately 41.3% were freshmen, and participants represented a wide range of academic majors; 6.5% were nursing majors, whereas 93.5% were non-nursing majors. In total, 28.3% of participants reported having a religious affiliation. On average, participants spent $156.1±132.3 per month on alcohol-related expenses and reported initiating alcohol consumption at a mean age of 18.9±2.9 years. In addition, 10.9% of participants were current smokers. Homogeneity testing of general characteristics and baseline outcome variables between the intervention and control groups revealed no statistically significant differences, indicating that the two groups were comparable at baseline (Table 1).

### Effects of the intervention on AUDIT score, TPB variables, alcohol outcome expectancies, and drinking refusal self-efficacy

Table 2 presents an analysis of the differences in mean values and changes from baseline between the intervention and control groups. Significant between-group differences in mean change scores were observed for positive attitudes toward drinking during pregnancy (t=−2.59, *p*=.013) and intentions to abstain from drinking during pregnancy (t=2.35, *p*=.005), with the intervention group demonstrating greater changes than the control group. Repeated-measures ANOVA revealed a significant group×time interaction for both attitude (F=6.69, *p*=.013) and intention (F=8.58, *p*=.005). These findings indicate that female college students in the intervention group experienced greater improvements in attitudes and intentions from baseline to the 4-week follow-up compared with those in the control group. In addition, a significant between-group difference was observed in drinking refusal self-efficacy following the intervention (t=2.47, *p*=.017).

## Discussion

The current study evaluated the efficacy of a web-based brief intervention aimed at reducing alcohol consumption among women of reproductive age. Previous studies have suggested that women in this age group should be evaluated at least annually for patterns of alcohol consumption or risky drinking behaviors [34]. These studies also emphasized the importance of providing appropriate counseling to prevent the adverse effects of alcohol consumption during pregnancy. Importantly, modifying drinking behavior before conception is essential to avoid prenatal alcohol exposure and associated fetal risks [35].

The intervention produced a significant effect on intention to abstain from drinking during pregnancy, consistent with previous research applying the TPB to alcohol use during pregnancy, which identified intention as the strongest determinant of drinking behavior during pregnancy [28]. Accordingly, it is reasonable to anticipate that increased intention to abstain from drinking during pregnancy, fostered by the web-based brief intervention developed in this study, may lead to reduced alcohol consumption in future pregnancies. It should be acknowledged, however, that intentions do not always translate directly into behavior, particularly when the behavior concerns a future life event that may not be imminent. Nevertheless, this study is meaningful in that it increased awareness among female college students—many of whom had not previously considered pregnancy or childbirth—regarding their current drinking behaviors and future reproductive health.

Our findings further indicate that the web-based brief intervention improved attitudes toward drinking during pregnancy and suggested a potential benefit for drinking refusal self-efficacy. These results are consistent with prior studies. For example, an intervention using TPB-based messaging to reduce alcohol consumption among university students resulted in improvements in attitudes, intentions, and self-efficacy [15]. Similarly, a study conducted in England demonstrated that college students exposed to TPB-based messages showed changes in intentions, attitudes, descriptive norms, and self-efficacy related to binge drinking [36].The observed changes in attitudes and intentions in the present study may be explained by the strong association between attitude and intention in women’s drinking behavior, as women are often more influenced by personal perceptions, such as attitudes, than by social norms [14]. Therefore, modifying personal beliefs may be particularly important when designing interventions to influence women’s drinking behavior.

The significant group-by-time interaction effects observed for both attitudes and intentions provide further insight into the intervention’s impact. Compared with the control group, which exhibited minimal change over time, participants in the intervention group demonstrated meaningful improvements from baseline to 4 weeks after the intervention. These findings suggest that the brief web-based program was effective not only in increasing awareness but also in sustaining cognitive and motivational changes over the short term. This pattern of improvement underscores the potential utility of web-based interventions in promoting behavioral change, particularly within the context of preconception health.

Although intention to abstain from drinking during pregnancy increased, AUDIT scores, which reflect current alcohol consumption, did not change significantly following the intervention. Both the intervention and control groups showed decreases in AUDIT scores over time, which may be attributable to government-imposed restrictions on private gatherings during the coronavirus disease-2019 pandemic. Previous reports have indicated that the frequency and volume of alcohol consumption among Korean college students declined during the pandemic, despite an increase in solitary drinking at home [37]. In addition, no significant changes were observed in alcohol outcome expectancies after the intervention. This may be because the outcome expectancies measured reflected general alcohol consumption rather than pregnancy-specific contexts, whereas the intervention emphasized negative birth outcomes as a deterrent to drinking during pregnancy. To obtain more precise insights in future research, the development of a measurement tool for pregnancy-specific alcohol outcome expectancies may be warranted.

The absence of significant intervention effects on subjective norms is consistent with findings from prior review studies examining social norms–based alcohol interventions among college students [38]. However, recent research suggests that exposure to alcohol-related content on social networking services can influence drinking behavior through subjective norms [39]. Accordingly, future research should explore innovative approaches, such as leveraging social media exposure, to more effectively influence subjective norms related to alcohol consumption among college students.

Several limitations of this study should be considered. First, the study assessed only intention to abstain from drinking during pregnancy; therefore, longitudinal research is needed to determine whether the web-based brief intervention leads to sustained changes in actual drinking behavior. Second, as a quasi-experimental study, the absence of random assignment may have introduced selection bias and unmeasured confounding, potentially limiting internal validity. However, baseline comparisons showed no significant differences in general characteristics between the intervention and control groups, suggesting that major confounders were statistically controlled. Another limitation relates to the use of a free downloadable drinking diary application and the Zoom platform for intervention delivery, which restricted the researcher’s ability to monitor participant engagement, track individual progress, or facilitate peer interaction. Although efforts were made to provide guidance and minimize barriers to using these tools, unmeasured biases may have occurred. Future studies should consider developing integrated web-based platforms that enable real-time engagement monitoring and facilitate peer communication. Finally, although self-report measures of drinking behavior are generally reliable and valid when confidentiality is ensured [40], response bias leading to underestimation of alcohol consumption remains possible.

Despite these limitations, the findings of this study have important implications for preconception care and the prevention of FASDs. The results support the need to educate reproductive-aged female college students—particularly those engaging in high-risk drinking—about alcohol prevention as a component of preconception nursing care. Moreover, the observed improvements in psychological constructs such as intention, attitude, and self-efficacy suggest that such interventions may be valuable even before observable behavior change occurs, especially when addressing preventive health behaviors among populations not actively considering pregnancy.

This study also suggests that a single 30-minute web-based brief intervention may serve as a practical tool for healthcare providers. Compared with traditional face-to-face interventions, web-based programs offer advantages including lower implementation costs, broader accessibility regardless of time and location, and enhanced scalability [41]. Given that college students tend to respond favorably to brief interventions with demonstrated effectiveness—whether delivered by counselors or through computer-based personalized feedback—web-based approaches may be particularly suitable for this population [42]. Additionally, the anonymity afforded by web-based platforms may promote more open reflection and engagement, especially when addressing sensitive issues such as alcohol use [43]. Accordingly, this study highlights the feasibility and potential effectiveness of web-based brief interventions as a flexible strategy for promoting preconception health among young women. Although the present study focused on female college students, replication in other populations, such as adolescents or women actively planning pregnancy, could further broaden the applicability of this intervention.

In conclusion, the brief web-based intervention led to significant improvements in attitudes toward drinking during pregnancy, intentions to abstain from drinking during pregnancy, and drinking refusal self-efficacy. Prioritizing the modification of current drinking behaviors is essential, as these behaviors directly affect women’s health and the health of future offspring. Simple, evidence-based interventions may therefore play a critical role in delivering effective preconception nursing care.

## Figures and Tables

**Table 1.** Homogeneity testing of general characteristics and outcome variables between the two groups at baseline

Characteristic	Categories	Total	n (%) or mean±SD		t or χ^2 ^(*p*)
			Experimental group (n=23)	Control group (n=23)	
General characteristic					
Age (year)		20.98±1.98	20.91±1.81	21.04±2.18	–0.22 (.826)
Year in college	Freshman	19 (41.3)	10 (43.5)	9 (39.1)	0.56 (.905)
	Sophomore	9 (19.6)	5 (21.7)	4 (17.4)	
	Junior	8 (17.4)	4 (17.4)	4 (17.4)	
	Senior	10 (21.7)	4 (17.4)	6 (26.1)	
Academic major	Nursing	3 (6.5)	2 (8.7)	1 (4.3)	0.36 (.552)
	Non-nursing	43 (93.5)	21 (91.3)	22 (95.7)	
Religion	Yes	13 (28.3)	6 (26.1)	7 (30.4)	0.11 (.743)
Expenditure on alcohol per month ($)		15.61±13.23	13.30±10.63	17.91± 15.29	–1.19 (.242)
Age at drinking onset (year)		18.87±2.88	19.30±1.52	18.43±3.78	1.02 (.311)
Smoking	Yes	5 (10.9)	4 (17.4)	1 (4.4)	2.02 (.155)
Outcome variable	Possible range				
AUDIT score	(0–40)	18.39±7.38	18.30±7.34	18.48±7.59	–0.08 (.937)
Attitude	(4–20)	5.57±2.14	5.78±2.37	5.35±1.90	0.69 (.496)
Subjective norms	(3–15)	13.02±1.87	13.26±1.84	12.78±1.91	0.87 (.391)
Intention	(3–15)	11.96±1.78	11.52±1.81	12.39±1.67	–1.69 (.097)
Alcohol outcome expectancies	(19–95)	64.91±11.63	64.21±11.79	65.61±11.69	–0.40 (.690)
Drinking refusal self-efficacy	(7–35)	19.91±5.94	20.61±6.22	19.22±5.70	–0.79 (.434)

AUDIT, Alcohol Use Disorders Identification Test.

**Table 2.** Comparison of outcome variables in the intervention and control groups before and after the intervention

Variable	Timepoint	Mean±SD		Between-group comparisons of scores			Between-group comparisons of changes from the baseline scores			F (*p*)
		Experimental group	Control group	Differences	95% CI	t (*p*)	Differences	95% CI	t (*p*)	
AUDIT score	Baseline	18.30±7.34	18.48±7.59	–0.17	–4.61 to 4.26	–0.08 (.937)				
	After 4 weeks	14.70±8.34	14.87±6.94	–0.17	–4.73 to 4.38	–0.08 (.939)	0	–2.14 to 2.14	0 (>.999)	
	Interaction (group ×time)									0 (>.999)
Attitude	Baseline	5.78±2.37	5.35±1.90	0.43	–0.84 to 1.71	0.69 (.496)				
	After 4 weeks	4.35±1.19	5.26±1.91	–0.91	–1.86 to 0.03	–1.94 (.058)	–1.35	–2.40 to 0.30	–2.59 (.013)	
	Interaction (group ×time)									6.69 (.013)
Subjective norms	Baseline	13.26±1.84	12.78±1.91	0.48	–0.63 to 1.59	0.87 (.391)				
	After 4 weeks	14.09±1.47	13.35±1.70	0.74	–0.2 to 1.68	1.58 (.122)	0.26	–0.67 to 1.19	0.57 (.573)	
	Interaction (group ×time)									0.32 (.573)
Intention	Baseline	11.52±1.81	12.39±1.67	–0.87	–1.90 to 0.16	–1.69 (.097)				
	After 4 weeks	12.87±1.87	12.35±1.80	0.52	–0.57 to 1.61	0.97 (.340)	1.39	0.43 to 2.35	2.35 (.005)	
	Interaction (group ×time)									8.58 (.005)
Alcohol outcome expectancies	Baseline	64.22±11.79	65.61±11.69	–1.39	–8.37 to 5.59	–0.40 (.690)				
	After 4 weeks	61.39±12.45	64.30±12.78	–2.91	–10.41 to 4.58	–0.78 (.438)	–1.52	–9.69 to 6.65	–0.38 (.709)	
	Interaction (group ×time)									0.14 (.709)
Drinking refusal self-efficacy	Baseline	20.61±6.22	19.22±5.70	1.39	–2.16 to 4.94	0.79 (.433)				
	After 4 weeks	23.83±6.42	19.35±5.73	4.48	0.86 to 8.10	2.47 (.017)	3.09	–0.26 to 6.43	1.86 (.070)	
	Interaction (group ×time)									3.81 (.057)

AUDIT, Alcohol Use Disorders Identification Test.
